# Exploring the dynamics of intentional sensorimotor desynchronization using phasing performance in music

**DOI:** 10.3389/fpsyg.2023.1207646

**Published:** 2023-11-02

**Authors:** Ji Chul Kim

**Affiliations:** Department of Psychological Sciences, Center for the Ecological Study of Perception and Action, Institute for the Brain and Cognitive Sciences, University of Connecticut, Storrs, CT, United States

**Keywords:** phasing, music performance, rhythmic coordination, coordination dynamics, oscillator model, dynamical systems

## Abstract

Humans tend to synchronize spontaneously to rhythmic stimuli or with other humans, but they can also desynchronize intentionally in certain situations. In this study, we investigate the dynamics of intentional sensorimotor desynchronization using phasing performance in music as an experimental paradigm. Phasing is a compositional technique in modern music that requires musicians to desynchronize from each other in a controlled manner. A previous case study found systematic nonlinear trajectories in the phasing performance between two expert musicians, which were explained by coordination dynamics arising from the interaction between the intrinsic tendency of synchronization and the intention of desynchronization. A recent exploratory study further examined the dynamics of phasing performance using a simplified task of phasing against a metronome. Here we present a further analysis and modeling of the data from the exploratory study, focusing on the various types of phasing behavior found in non-expert participants. Participants were instructed to perform one phasing lap, and individual trials were classified as successful (1 lap), unsuccessful (> 1 laps), or incomplete (0 lap) based on the number of laps made. It was found that successful phasing required a gradual increment of relative phase and that different types of failure (unsuccessful vs. incomplete) were prevalent at slow vs. fast metronome tempi. The results are explained from a dynamical systems perspective, and a dynamical model of phasing performance is proposed which captures the interaction of intrinsic dynamics and intentional control in an adaptive-frequency oscillator coupled to a periodic external stimulus. It is shown that the model can replicate the multiple types of phasing behavior as well as the effect of tempo observed in the human experiment. This study provides further evidence that phasing performance is governed by the nonlinear dynamics of rhythmic coordination. It also demonstrates that the musical technique of phasing provides a unique experimental paradigm for investigating human rhythmic behavior.

## 1. Introduction

Synchronization is a natural phenomenon found widely in both living and non-living systems (Pikovsky et al., 2001; Strogatz, 2003). Humans synchronize their movement to external rhythms seemingly effortlessly and automatically (Repp, 2005; Repp and Su, 2013), and interpersonal synchronization is fundamental to the coordination and communication in social interaction (Schmidt and Richardson, 2008; Shockley et al., 2009; Keller et al., 2014). Humans can also desynchronize intentionally under certain circumstances such as competitive sports (Yamamoto et al., 2013; McGarry and De Poel, 2016) and argumentative conversations (Paxton and Dale, 2013, 2017). Intentional desynchronization, however, is not a simple switch of behavior but involves complex dynamics because humans, despite the intention, tend to synchronize sometimes unknowingly. For example, a study of music performance in an Afro-Brazilian ritual showed that two independent groups playing different music synchronized unintentionally when they were in close proximity (Lucas et al., 2011). Intentional desynchronzation offers an interesting setup for studying human coordinative behavior, but little work has been done to study it systematically. Here, we aim to study the dynamics of intentional sensorimotor desynchronization in a controlled situation that originates from music performance, called *phasing*.

**Figure 1:**
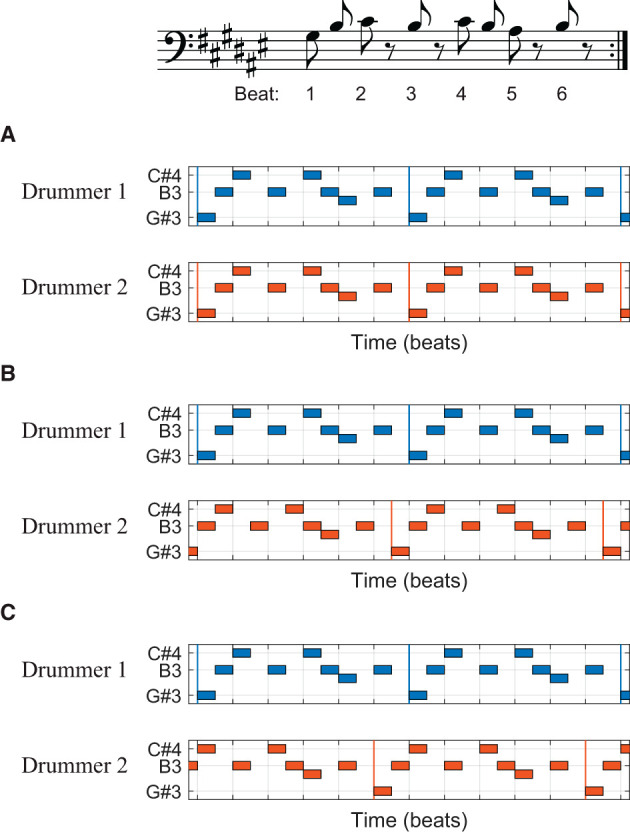
A phasing section from Steve Reich's Drumming (1971/2011). The basic six-beat pattern is shown in musical notation **(top)**, and different alignments of two drummers are shown in piano-roll representations **(A–C)**. Vertical colored lines indicate the beginning of each repetition of the pattern. **(A)** Two drummers' beats are aligned at the beginning of the phasing process. Drummer 2 is **(B)** a half beat (an eighth note) ahead of Drummer 1 in the middle and **(C)** one full beat (a quarter note) ahead at the end of phasing.

Phasing is a compositional technique in contemporary art music popularized by the composer Steve Reich. It is a process in which two identical patterns are played in and out of phase, with their relative phase (or phase difference) varying over time (Cohn, 1992; Yust, 2021). In Steve Reich's Drumming (1971/2011), two drummers start a phasing process by repeating the same six-beat pattern *in phase*, that is, with their performance aligned in time (Figure 1A). Then, they gradually desynchronize, with Drummer 2 (the moving part) increasing tempo slightly while Drummer 1 (the steady part) holds the original tempo, so that Drummer 2 is one quarter note ahead of Drummer 1 after about 20 or 30 s (Figure 1C; Reich, 1971/2011). Thus, according to the musical score, the relative phase between the musicians should increase linearly while phasing. Given that typical ensemble performance requires synchronization between musicians, phasing asks for an unusual (and somewhat unnatural and counterintuitive) skill of intentional desynchronization.

In a previous case study, two world-renowned percussionists performed the phasing section from *Drumming* (Hartenberger, 2016; Schutz, 2019). It was found that despite their intention to follow the musical score (i.e., one increases tempo while the other holds a constant tempo), both musicians sped up and slowed down together throughout the phasing process. A further analysis of the performance data revealed systematic nonlinear trajectories in the tempi and the relative phase (Kim, manuscript in preparation).^1^ The relative phase, instead of increasing in a steady rate, advanced in a series of plateaus and abrupt transitions. The relative phase plateaued when the combined rhythm formed a simple pattern, such as the interlocking pattern where the note onsets are aligned (Figure 1B) and the interleaved pattern where the onsets form a steady sixteenth-note stream (e.g., halfway between Figures 1A, B). The drummers increased tempo together while they were engaged in one of these stable patterns until their tempi eventually diverged, after which they quickly moved to the next stable pattern.^2^

The nonlinear trajectories found in the expert data suggest that phasing performance is governed by the nonlinear dynamics of rhythmic coordination. Previous research showed that the coordination of rhythmic movements is stable when individual movements are arranged in either in-phase or antiphase relationship (Kelso, 1984, 1995). This means that the coordinated movements, when described as a dynamical system (Strogatz, 1994), have attractors at the in-phase and the antiphase states (Haken et al., 1985; Kelso, 2008). For the phasing performance in *Drumming*, the interlocking patterns (Figures 1A–C) and the interleaved patterns (halfway between the interlocking patterns) serve as attractors because the beats played by two musicians are either aligned (in-phase) or interleaved (antiphase) in these patterns. When one drummer increases tempo while locked in one of these stable patterns, the other drummer is also inclined to increase tempo involuntarily (and unknowingly) due to the stability and attraction of the coordinated pattern. Hence, the nonlinear trajectories found in phasing performance can be interpreted as resulting from the dynamic interaction between the intention of desynchronization (phasing) and the involuntary tendency of synchronization. This dynamical systems interpretation was supported by a model of two mutually coupled oscillators, which showed similar nonlinear trajectories to those found in the expert performance (Kim, in preparation).

The above case study suggests that phasing performance offers a unique window into the dynamics of human rhythmic coordination (see also Van Kerrebroeck et al., 2021, for an interesting study of phasing in virtual reality) and this led us to the idea of using phasing as an experimental paradigm. We conducted an exploratory study with non-expert participants (i.e., no professional musicians) who performed phasing against a metronome by finger tapping (Hall et al., 2023). The goal of the study was to observe a wide range of phasing behaviors beyond what is seen in the highly controlled performance of expert musicians. Since phasing with a human partner is difficult even for trained musicians (see Hartenberger, 2016, for suggestions for practicing phasing) we began the investigation with a simplified task of phasing against a metronome. This simplifies the task in two ways: Phasing is performed (1) with a non-responsive partner who does not react to the participant's tempo change, and (2) using a simple isochronous rhythm rather than a complex rhythmic pattern (such as the one in Figure 1). We reported elsewhere the results of multidimensional recurrence quantification analysis of the relative phase data (Hall et al., 2023). The analysis showed that tapping was more stable when the taps were near in-phase or antiphase relation with the metronome, confirming the existence of in-phase and antiphase attractors in phasing performance.

In the present paper, a further analysis and modeling of the data from the exploratory study (Hall et al., 2023) is reported. Here we examine various types of phasing behavior observed in non-expert participants and analyze the effect of metronome tempo which was the only parameter systematically varied in the study. It will be shown that successful phasing depends on gradual advancement of each tap relative to the metronome. This finding, along with the effect of tempo on phasing performance, is replicated in a dynamical model consisting of an adaptive-frequency oscillator coupled to a periodic stimulus. To help the readers, a brief version of the experimental procedure is given below (see Hall et al., 2023, for the original description).

## 2. Materials and methods

### 2.1. Participants and materials

Twenty-five undergraduate students (17 females, 8 males; 18–21 years, *M* = 18.7 years) at the University of Connecticut were recruited from the Department of Psychological Sciences Experiment Participant Pool and received course credit for participating in the experiment. Nineteen out of 25 participants (12 females, 7 males) reported no experience in playing musical instrument. Six participants (5 females, 1 male) reported 1 to 13 years of experience in playing musical instrument(s), but none of them were music-major students.

Seven audio stimuli were created, each containing a series of identical metronome beats (woodblock sound) at a constant tempo, ranging from 80 BPM (beats per minute) to 140 BPM in 10-BPM increments. Each stimulus was 2 minutes long and contained a bell sound (serving as a cue signal) coinciding with a metronome beat at around 7 s after the first metronome beat.

### 2.2. Procedure

**Figure 2:**
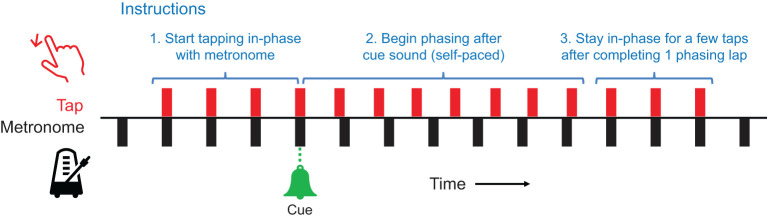
The procedure of a phasing trial.

The participants first filled out a demographic survey including the length of experience in playing musical instruments (in years). Then, the experimenter gave the instructions for performing phasing against a metronome, accompanied by audio and audiovisual demonstrations (audio: metronome and tapping sounds; audiovisual: a video of a person performing phasing in the same experimental setup). The participants were instructed (Figure 2) to (1) start tapping to the metronome using a finger of their choice and maintain in-phase (synchronous) tapping, and (2) upon hearing the cue signal (bell sound), begin phasing in a steady but self-paced manner by placing each tap increasingly ahead of the metronome.^3^ (3) Once they completed one phasing lap by coming back to in-phase tapping, they were asked to tap a few more times in phase and stop (the audio stimulus stopped 5 seconds after tapping stopped).

The participants then practiced phasing with an interactive audiovisual display (Practice 1). The visual display included an arrow on a circle (like a clock face) which indicated the relative phase of each tap, with in-phase (relative phase = 0) at 12 o'clock and antiphase (±π or 180°) at 6 o'clock. Thus, the goal was to make the arrow move clockwise from 12 o'clock and stop when it comes back to 12 o'clock after one lap. The number of phasing laps (i.e., the number of clockwise rounds made by the arrow) was displayed on top of the display. After each practice trial, feedback was given on the screen about the number of phasing laps made in total (the target was one lap) and a score indicating whether the phasing was gradual enough. The score of 100% was given when the participant made at least 16 taps while phasing. This excluded initial and final in-phase taps. This minimum required number of taps was not known to the participants to encourage self-paced tapping. The minimum number of phasing taps was not used in data analysis. In Practice 2, the same feedback was given on the screen after each trial, but no visual aid was provided. In both practice sessions, three metronome tempi (90, 110, and 130 BPM) were used to prepare the participants for the range of tempo used in the main experiment (80–140 BPM).

The main experiment included 21 trials, three trials for each of 7 metronome tempi. Metronome tempo was the only independent variable in this exploratory study. The order of the trials was pseudo-randomized for each participant such that the same tempo was not presented in any two consecutive trials. No feedback was provided during the main experiment. The audio stimuli were played at a comfortable listening level through two loudspeakers placed in front of the participant. The participants tapped their finger on HandSonic HPD-15 Hand Percussion Pad (Roland Corporation), and the MIDI signal was converted to keystrokes using MIDI Translator Pro (Bome Software GmbH & Co. KG). Hitting on the drum pad created an audible thud, and no additional sound was played in response to tapping. The timestamp of each tap was recorded with custom code written in MATLAB (MathWorks, Inc.) using Psychophysics Toolbox (Psychtoolbox) Version 3.0.15.

### 2.3. Analysis

The relative phase of the *n*th tap, *ψ*_*n*_, was calculated from the timestamp of the tap, *t*_*n*_, and the timestamp of the closest metronome beat, *m*_*n*_, by


(1) $$\begin{array}{l} \psi_{n}=\frac{2\pi \left(m_{n}-t_{n}\right)}{T}, \end{array}$$

where *T* is the period of the metronome. Thus, the relative phase ranged from −π to π (before upwrapping, see below), and a positive relative phase resulted when the tap preceded the metronome beat.^4^ The time series of relative phases in an individual trial were then unwrapped (using MATLAB's unwrap function) so that the relative phase can increase continuously beyond π toward 2π, instead of jumping down to −π. This allowed counting the number of phasing laps as described below.

**Figure 3:**
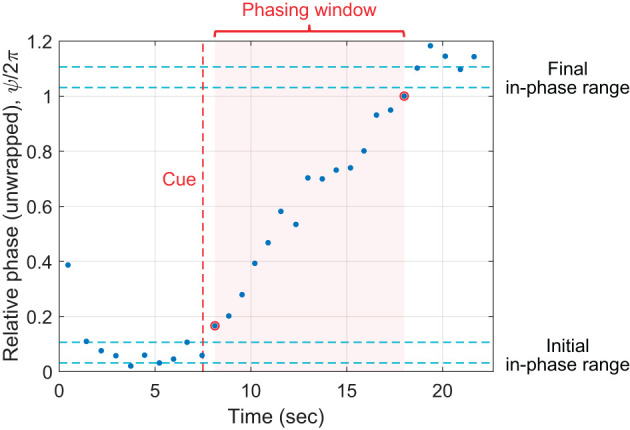
Parsing of a phasing trial. The unwrapped relative phase of each tap is plotted vs. its timestamp. The vertical axis displays unwrapped relative phase divided by 2π to show the number of phasing laps clearly. In this particular trial, one phasing lap was made, with 15 taps within the phasing window. See text for details.

From the relative phase data for each trial, we determined (1) the number of phasing laps made (i.e., the number of 2π-rounds made by the relative phase), and (2) the number of taps made during phasing (i.e., taps made between initial and final in-phase tapping). We first determined the range of relative phase during initial in-phase tapping by taking the minimum and the maximum relative phase during the second half of the pre-cue interval (see Figure 3). The initial range was determined for each trial because it is known that the mean asynchrony between taps and metronome beats during in-phase synchronization varies across individuals and also depends on tempo (Aschersleben, 2002; Scheurich et al., 2018). The number of phasing laps was calculated as the number of complete 2π-rounds made between the minimum initial relative phase and the maximum unwrapped relative phase in the trial.^5^ The number of taps during phasing was determined by identifying the phasing window, which begins after the last tap inside the initial in-phase range and ends before the first tap inside the final in-phase range (indicated by the pink background in Figure 3).

## 3. Results and discussion

### 3.1. Successful vs. unsuccessful trials: gradualness of phasing

#### 3.1.1. Classification of phasing outcomes

**Figure 4:**
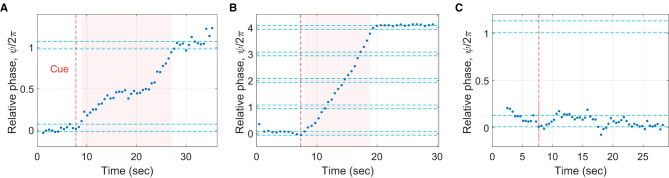
Examples of different phasing outcomes. **(A)** A successful trial (one phasing lap made as instructed), **(B)** an unsuccessful trial (more than one phasing lap; 4 laps were made in this particular trial), and **(C)** an incomplete trial (no complete phasing lap made).

A phasing trial was counted as “successful” if the participant was able to follow the instructions and perform only one phasing lap (Figure 4A). Only 38% of all trials (200 out of 525 trials) were successful trials, indicating the difficulty of the phasing task. The trials with more than one phasing lap were named “unsuccessful”, and they accounted for 41% of all trials (213 trials; Figure 4B). The trials with no complete phasing lap were labeled “incomplete”, accounting for 19% of all trials (102 trials, Figure 4C). The trials in which the participant did not maintain in-phase tapping before the cue signal were flagged as “noncompliant” (the remaining 2%, 10 trials) and excluded from subsequent analysis because initial in-phase range could not be determined.^6^

#### 3.1.2. Gradualness of phasing: number of taps per phasing lap

**Figure 5:**
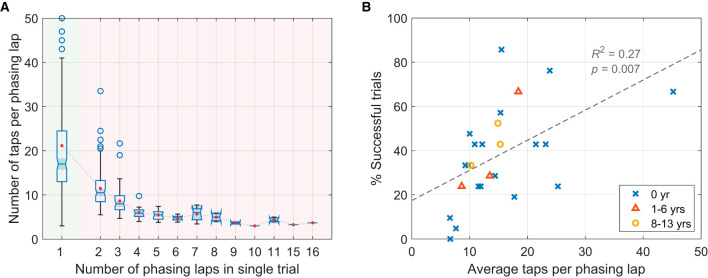
**(A)** Box plots showing the number of taps per phasing lap for trials grouped by the total number of phasing laps (green background: successful trials, red background: unsuccessful trials). The red dots show the mean taps per lap for each group. Nine trials with more than 50 taps/lap (the max at 84 taps, all successful trials) are beyond the plotting range and not shown. **(B)** Success rate (the percentage of successful trials) for individual participants (*N* = 25) plotted against their average taps per phasing lap (for the successful and the unsuccessful trials combined). Participants with different levels of musical experience (in years of playing musical instruments) are indicated by different markers (see the legend).

A major difference found between the successful and the unsuccessful trials was the gradualness of phasing, which was quantified by the number of taps per phasing lap (only the taps in phasing window were counted, excluding initial and final in-phase taps). A high number of taps per lap indicates small increments of relative phase by individual taps on average. It was found that significantly fewer taps were made per phasing lap in the unsuccessful trials (*M* = 8.77, *SD* = 4.44) than in the successful trials (*M* = 21.15, *SD* = 13.16), *t*(241.2) = 12.65, *p* < .001, 95% CI [10.45, 14.31] (Welch's two sample *t*-test).^7^ The box plots in Figure 5A show the distribution of the number of taps per lap in individual trials grouped by the number of phasing laps made. Note that the shaded notch for the successful trials (one phasing lap, green background) does not overlap with the notches for the unsuccessful trials (2 or more laps, red background). This indicates that the median number of taps per lap was greater in the successful trials than in the unsuccessful trials at a 95% confidence level (McGill et al., 1978).

The same relationship was also found at the level of individual participants. The average number of taps per phasing lap for individual participants predicted the percentage of successful trials, *R*^2^ = 0.275, *F*_(1,23)_ = 8.70, *p* = 0.007 (Figure 5B). However, the years of playing musical instruments (indicated by different markers in Figure 5B) did not predict the success rate, *R*^2^ = 0.028, *F*_(1,23)_ = 0.658, *p* = 0.426.

### 3.2. Effect of tempo

#### 3.2.1. Phasing outcome types by tempo

**Figure 6:**
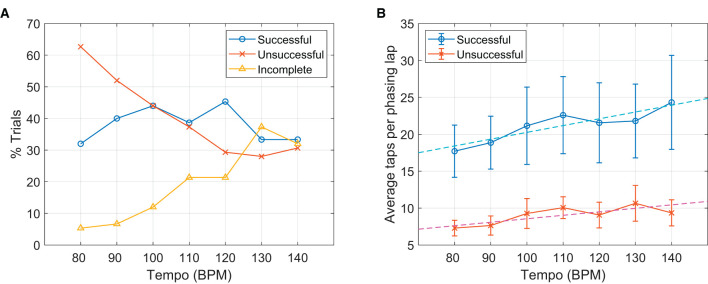
Effect of metronome tempo. **(A)** The proportions of three different phasing outcomes for each metronome tempo. **(B)** Average taps per phasing lap in the successful and the unsuccessful trials shown for each tempo. Error bars indicate 95% confidence intervals, and dashed lines show the linear fits for the successful and the unsuccessful trials separately.

Figure 6A shows that the number of successful trials did not vary significantly with the tempo, *r*(5) = −0.11, *p* = 0.807, but clear and opposite trends were found for the unsuccessful and the incomplete trials. The number of unsuccessful trials decreased as the tempo increased, with a strong negative correlation, *r*(5) = −0.94, *p* = 0.002, whereas the number of incomplete trials was positively correlated with the metronome tempo, *r*(5) = 0.95, *p* = 0.001. This indicates different types of failure were prevalent at slow vs. fast tempi. At slow metronome tempi, the participants often failed to stop at the in-phase target after one phasing lap. At faster tempi, on the other hand, more participants were not able to reach the in-phase target.

#### 3.2.2. Number of taps per lap by tempo

An effect of metronome tempo was also found in the number of taps per phasing lap. For both the successful and the unsuccessful trials, phasing was more gradual (i.e., more taps per lap) at higher tempi (Figure 6B). A multiple linear regression indicated that outcome type (successful = 1, unsuccessful = 2) and metronome tempo explained a significant portion of the variance in the taps/lap data, *R*^2^ = 0.30, *F*_(2,410)_ = 89.4, *p* < 0.001. Both outcome type (*B* = −12.03, *t* = −12.58, *p* < 0.001) and metronome tempo (*B* = 0.068, *t* = 2.80, *p* = 0.005) were significant predictors in the model. This finding might be related to the decrease of unsuccessful trials and the increase of incomplete trials with increasing tempo shown above (Figure 6A). Gradual phasing at fast tempi may reduce the chance of overshooting and skipping over the goal, but at the same time, it may make it more difficult to leave the initial in-phase tapping. Although this did not change the success rate across tempi, this might have impacted the composition of failed trials (i.e., unsuccessful vs. incomplete). We discuss this possibility further in Section 4.1.

### 3.3. Subtypes of incomplete trials

**Figure 7:**
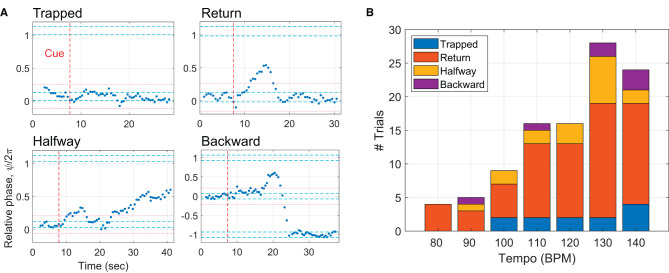
**(A)** Subtypes of incomplete trials: trapped, return, halfway, and backward. The magenta dotted lines indicate the initial in-phase range ± one width of the range, which was used to determine trapped trials. **(B)** The number of the subtypes of incomplete trials shown for each metronome tempo.

The above finding encouraged closer examination of the incomplete trials as to how phasing failed when no complete phasing lap was made. Multiple subtypes of the incomplete trials were identified (Figure 7A). In the first subtype named “trapped”, taps did not leave the initial in-phase range significantly. A trial was determined to be trapped if the relative phases of all taps after the cue signal were inside the initial range plus and minus one width of the range (indicated by the magenta dotted lines). A second subtype is called “return” because taps leave the initial in-phase range but return to it without reaching the goal. In a third subtype, taps leave the initial range but stop before reaching the goal, hence called “halfway”. The last subtype is called “backward”, in which taps leave the initial range but go in the wrong direction. Figure 7B shows that return trials were the most common subtype of incomplete trials for all tempi. It is noteworthy that all incomplete trials at the lowest tempo (80 BPM) were return trials and that trapped trials were found only at the mid to high tempi.

## 4. Dynamical systems modeling

### 4.1. Dynamical systems account of the experimental results

Unlike the professional percussionists in the expert study (Hartenberger, 2016; Schutz, 2019), the non-expert participants in the exploratory study further analyzed here (Hall et al., 2023) often failed to perform phasing as instructed (in unsuccessful and incomplete trials). By recruiting non-expert participants, we hoped to observe a diverse range of phasing behaviors (especially failures), which can reveal more about the underlying dynamics of phasing performance than the highly controlled performance of expert musicians does. Here we discuss the findings of the exploratory study from a dynamical systems perspective, attempting to characterize the various phasing outcomes observed in the experiment as different possible behaviors of a single dynamical system. The ideas developed here are tested with model simulations below.

**Figure 8:**
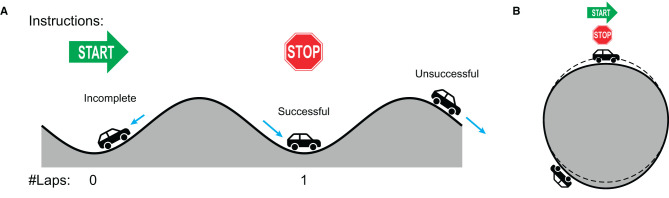
A car analogy for the dynamic landscape of phasing performance. **(A)** An unwrapped flat representation. The in-phase attractor is depicted as a valley. **(B)** The true circular landscape where phasing starts and ends at the same point. The dashed line depicts a perfect circle so that it is a valley if the ground is below the dashed line and a hill if above the dashed line.

Dynamical systems theory describes the behavior of complex dynamical systems with mathematical equations which capture the contribution and interaction of underlying forces and constraints (Strogatz, 1994; Kelso, 1995; Schiavio et al., 2022). As was done for phasing between human partners (see Section 1), the dynamics of phasing against a metronome can be characterized in terms of the interaction between the intrinsic tendency of synchronization and the intention of desynchronization. In-phase coordination is an intrinsically stable mode of rhythmic coordination,^8^ so that a coordinated rhythmic movement such as finger tapping to a metronome is attracted to the in-phase state when the current state is near it (Kelso, 1984; Scholz et al., 1987). To perform phasing, however, the tapper has to overcome the attraction of in-phase coordination by increasing tapping tempo and place each tap increasingly ahead of the metronome. This requires a precise control of tapping tempo because if the tempo increase is not big enough, taps would be pulled back toward the metronome beats and hence unable to escape the in-phase attractor, as found in some of incomplete trials. To use an analogy, this is like a car trapped in a valley and unable to climb up the hill because it cannot overcome the gravity (the car labeled “Incomplete” in Figure 8A).

If the tempo increase is too big, on the other hand, the tapper would be able to escape the initial in-phase attractor but would also be prone to miss the target by overshooting. An unsuccessful trial can be depicted as a system with high kinetic energy enough to escape the origin attractor but is unable to stop at the goal attractor (like the car labeled “Unsuccessful” in Figure 8A). This interpretation agrees with the finding that fewer taps per phasing lap were made in the unsuccessful trials (Figure 5), which indicates that the participants tended to miss the target and kept phasing when they increased tapping tempo too much.

Successful phasing, however, requires more than just the precise amount of tempo increase. The control of tapping tempo has to be context-dependent because the attractor one wants to escape from (i.e., in-phase coordination) is the same attractor one wants to land on (Figure 8B). Hence, tapping has to accelerate to start phasing and decelerate when finishing, just as the “Successful” car in Figure 8A must accelerate uphill and then decelerate downhill to stop at the stop sign.

Another important finding that must be addressed in the dynamical systems account is the effect of metronome tempo. It was found that unsuccessful trials were more common at slow tempi, while incomplete trials were found more often at fast tempi (see Figure 6A). One possible interpretation that follows from the dynamical systems account is that the attraction of in-phase coordination might be stronger at fast tempi. A strong attractor would be harder to escape, resulting in more incomplete trials, whereas a weak attractor would be easy to escape but also easy to overshoot, leading to more unsuccessful trials. This idea seems consistent with the finding that more taps were made per phasing lap at faster tempi (Figure 6B). The same amount of tempo increase would result in a slower advancement of relative phase when attempting to escape a stronger attractor (imagine a rocket launch from a heavy planet).

But why would the in-phase attractor be stronger at fast tempi? A clue can be found in the experiments of sensorimotor synchronization in the presence of distractors (Repp, 2003, 2004). In these experiments, participants were instructed to synchronize finger taps with a target sequence of isochronous tones while ignoring a sequence of distractor tones which were presented at a different pitch. The results showed that the taps were attracted toward the distractor tones when they were close in time. Repp (2004) found that the strength of distractor effect depended on the absolute temporal separation between the target and the distractor (measured in milliseconds, for example) rather than the relative phase between them (e.g., in radian). In dynamical systems language, this means that the temporal basin of attraction for in-phase coordination, where taps are pulled toward metronome beats, may have a fixed width when measured in absolute time regardless of metronome tempo. Then, the basin of in-phase attraction would take up a bigger portion of the metronome period when the tempo is fast (because the period is short), and this could result in stronger in-phase attraction at fast tempi. We test this idea below with a dynamical model of phasing performance.

Our goal is to construct a minimal dynamical model capable of producing various phasing behaviors observed in the human participants and test if the model can replicate the main findings of the experiment. We start with a simple, well-known model of periodically forced oscillation and discuss additional dynamical features necessary to model human phasing behaviors (Section 4.2). We then present a minimal model with such features and test if it can replicate the experimental results (Section 4.3).

### 4.2. Model with fixed frequency detuning

Systems showing periodic activities, such as flashing fireflies and the human sleep-wake cycle, can be modeled as oscillations (Pikovsky et al., 2001; Winfree, 2001), and human rhythmic movement such as periodic finger tapping has been studied with oscillator models (Haken et al., 1985; Large and Kolen, 1994; Large et al., 2015). Let us consider a phase oscillator coupled to a periodic external stimulus as a model of phasing performance against a metronome,


(2) $$\begin{array}{l} \frac{d\phi }{dt}=\omega +c\sin\left(\theta -\phi \right), \end{array}$$

where *ϕ* is the oscillator phase,^9^ *t* is time, *ω* is the oscillator's natural frequency, *c* is the coupling strength, and *θ* = *ω*_0_*t* is the stimulus phase where *ω*_0_ is the stimulus frequency (hence we assume *θ* = 0 at *t* = 0 without loss of generality). In the absence of external stimulus (i.e., when *c* = 0), *ϕ* increases at the constant rate of *ω*. (Hence, the phase *ϕ* obtained by integrating the differential equation, Equation (2), is an unwrapped phase, which is not confined to a 2*π*-range.) We assume that the oscillator produces a “tap” whenever the wrapped *ϕ* crosses zero^10^ (or when the unwrapped phase crosses a multiple of 2*π*), an arbitrary choice that does not alter the overall results. Similarly, the stimulus is assumed to produce a “tick” (like a metronome) whenever the wrapped *θ* crosses zero.

**Figure 9:**
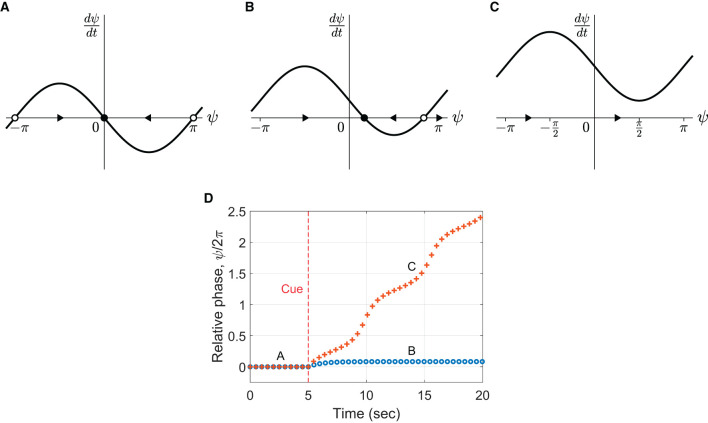
Flow in the vector field defined by Equation (3) for **(A)** Δ*ω* = 0, **(B)** Δ*ω* = 0.5, and **(C)** Δ*ω* = 1.5 for *c* = 1. The arrows indicate the direction of flow. The filled and empty circles denote stable and unstable fixed points, respectively. **(D)** Simulations of the model with fixed detuning (Equation 2) with the parameters in **(B, C)**. Frequency detuning is introduced at *t* = 5 sec. The markers indicate the (unwrapped) relative phase of individual “taps” produced by the oscillator (phase zero-crossing). Common parameter: *ω*_0_ = 4*π* rad/sec (i.e., 2 Hz or 120 BPM).

The synchronization and desynchronization of the oscillator with the external stimulus can be described by the relative phase defined as *ψ* = *ϕ*−*θ*. Thus, *ψ* is positive when the oscillator is ahead of the stimulus, as defined for the experimental data (see Equation 1). Since *d*θ**/*dt* = *ω*_0_, the relative phase is governed by the differential equation,


(3) $$\begin{array}{l} \frac{d\psi }{dt}=\Delta \omega -c\sin\psi , \end{array}$$

where Δ*ω* = *ω*−*ω*_0_ is *frequency detuning*, the difference between the oscillator's natural frequency and the stimulus frequency. This equation represents a vector field that determines the flow of *ψ* over time (Figures 9A–C, the direction of flow is indicated by arrows). The points where *d*ψ**/*dt* = 0 are called fixed points because *ψ* does not change over time at these values (indicated by circles). If the local flow is toward a fixed point, it is called a stable fixed point or an attractor (filled circles). If the flow is away from a fixed point, it is called an unstable fixed point or a repeller (empty circles).

When Δ*ω* = 0 (no frequency detuning), there is a stable fixed point at *ψ* = 0 (Figure 9A), which indicates that *ψ* converges to 0 over time. Thus, the oscillator synchronizes in phase with the stimulus when its natural frequency matches the stimulus frequency. In the presence of frequency detuning, the oscillator may or may not synchronize with the stimulus depending on the size of detuning. When the detuning is small compared to the coupling strength (|Δ*ω*| < *c*, Figure 9B), *ψ* converges to a nonzero steady-state value, indicating that the oscillator synchronizes (or phase-locks) with the stimulus maintaining a constant nonzero relative phase. When the frequency detuning is larger than the coupling strength (|Δ*ω*|>*c*, Figure 9C), no fixed point exists, and the flow is in one direction which depends on the sign of Δ*ω*. This indicates that the oscillator desynchronizes with the stimulus when the detuning is large enough. (See Section 4.5 of Strogatz, 1994, for a more detailed and reader-friendly analysis of the model).

The above analysis suggests that different phasing behaviors may be simulated by manipulating the model parameters Δ*ω* and *c*. Figure 9D shows two simulations with the different amounts of detuning shown in Panels B and C. To simulate in-phase tapping at the beginning of a phasing trial, the natural frequency was first set identical to the stimulus frequency (i.e., Δ*ω* = 0, Panel A). After the cue signal at 5 s, the natural frequency was increased (i.e., detuned) by a fixed amount to simulate the intention of phasing. When the frequency increase was small (Δ*ω* < *c*, the circle markers in Panel D), the oscillator did not perform phasing but remained phase-locked to the stimulus with a positive relative phase. In other words, the oscillator “tapped” ahead of the metronome but could not escape the influence of the in-phase attractor. This behavior corresponds to the incomplete trials found in the human experiment. When the frequency increase was larger than the coupling strength (Δ*ω*>*c*, the + markers), the oscillator desynchronized with the stimulus. However, it did not resynchronize after one phasing lap but continued phasing because the large frequency detuning had eliminated the attractor (Figure 9C). This behavior corresponds to the unsuccessful trials. Thus, the simulations show that the model with a fixed amount of frequency detuning can produce only the incomplete and the unsuccessful types of phasing behavior.^11^ The results suggest that a context-dependent control of frequency detuning is required to simulate successful phasing as discussed in the previous section.

### 4.3. Phasing model with context-dependent frequency dynamics

Based on the above observations, we expanded the simple oscillator model discussed above (Equation 2) with additional features. The resulting model is described by two differential equations,


(4) $$\begin{array}{ll} \frac{d\phi }{dt} & =\omega +cf\left(\phi ,\theta ,\rho \right), \end{array}$$

(5) $$\begin{array}{ll} \frac{d\omega }{dt} & =\gamma f\left(\phi ,\theta ,\rho \right)-\lambda g\left(\phi ,\theta ,\rho \right)\left(\dot{\psi }-\Delta \right), \end{array}$$

which determine the dynamics of the phase (*ϕ*) and the natural frequency (*ω*) of the oscillator, respectively. Here we present a synopsis of the model structure first (see Table 1 for a summary) and describe the details of each model component below (Section 4.3.1). (Readers may choose to skip the details and proceed to the replication results.)

**Table 1:** Components of the context-dependent phasing model (Equations 4–5).

Variables	*ϕ*	Oscillator phase
	*ω*	Oscillator natural frequency
	*θ*	Stimulus phase (external)
Parameters	*c*	Coupling strength (phase attraction)
	γ	Frequency adaptation rate
	λ	Frequency bias strength
	Δ	Target change rate of relative phase (controls gradualness of phasing)
	ρ	Parameter controlling the width of pulse-like functions
Functions	*f*	Pulse-like coupling function
	*g*	Context-dependent gating function (frequency bias weakens once the system escapes the in-phase attractor)

The phase equation (Equation 4) includes the natural frequency (*ω*) and the coupling term (strength *c*). The pulse-like function *f* is used as the coupling function (instead of the sine function in Equation 2) so that phase attraction is strong only when the tap and the metronome tick are close in time. We will keep the temporal width of the coupling function constant across different metronome tempi and test if this allows the model to replicate the tempo effect found in the human data as discussed above (Section 4.1).

The frequency equation (Equation 5) includes frequency adaptation (adaptation rate γ) and frequency bias (strength λ), which have opposing effects. The former causes the natural frequency to adapt to the stimulus frequency (promoting synchronization), while the latter causes the natural frequency to deviate (detune) from the stimulus frequency to keep the relative phase increasing at the constant rate of Δ (promoting desynchronization and phasing). The context-dependent gating function *g* manipulates the balance between the two terms to initiate and end phasing, by starting with strong frequency bias and switching to weaker bias once the system escapes the initial in-phase attractor. We will manipulate Δ (target change rate, which determines the gradualness of phasing) and γ (frequency adaptation rate) to replicate the multiple phasing outcomes found in the human experiment as well as the effect of metronome tempo on the composition of phasing outcome types.

#### 4.3.1. Detailed model description

**Figure 10:**
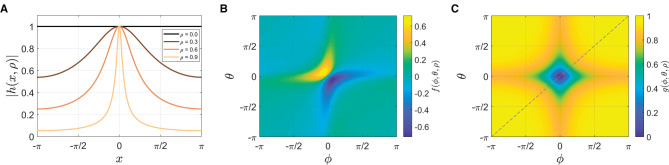
**(A)** The amplitude of pulse-like function *h*(*x*, ρ) for ρ= 0, 0.3, 0.6, and 0.9 (see Appendix for the equation). **(B)** The coupling function *f*(*θ*, *ϕ*, ρ) for ρ = 0.7 (Equation 6). **(C)** The gating function *g*(*θ*, *ϕ*, ρ) when *ψ* > *ψ*_*θ*_ for ρ = 0.7 (Equation 7). The diagonal indicates where the relative phase is zero (*ψ* = *ϕ* − *θ* = 0).

The phase equation of the context-dependent model (Equation 4) is identical to the simple model (Equation 2) except the coupling function. *f* is a pulse-like coupling function defined as


(6) $$\begin{array}{l} f\left(\phi ,\theta ,\rho \right)=\text{Im}\left[h\left(\theta ,\rho \right)\cdot h\left(-\phi ,\rho \right)\right], \end{array}$$

where Im[*x*] denotes the imaginary part of *x*, *h* is a complex analytic function with a pulse-like shape defined in Appendix, and *θ* = *ω*_0_*t* is the stimulus phase. The width of the pulse function *h*(*x*, ρ) depends on the parameter ρ, becoming sharper as ρ increases between 0 and 1 (Figure 10A; Appendix for more details). The coupling function *f* is a product of two pulse-like functions, one generated by the stimulus (*θ*) and the other by the oscillator (*ϕ*). This is a more realistic coupling function for modeling tapping to a metronome than sin(*θ*−*ϕ*) in Equation (2), given that the stimulus sound used in the experiment (a woodblock sound) is a discrete event and that taps are attracted toward stimulus tones when they occur in temporal proximity (Repp, 2003, 2004). Figure 10B shows that the coupling interaction is strong when both *θ* and *ϕ* are near zero, in other words, when the metronome beats and the taps are close in time.^12^ *f* can be considered a general coupling function that encompasses the sine coupling in Equation (2) because when ρ = 0, *f*(*ϕ*, *θ*, 0) = sin(*θ*−*ϕ*), that is, Equation (4) becomes Equation (2) when ρ = 0 (see Appendix).

The second differential equation of the model (Equation 5) describes the dynamics of the natural frequency of the oscillator *ω*, which is now made a variable (it was a parameter in the first model). The dynamics of *ω* is determined by two factors. The first term on the right-hand side introduces frequency adaptation, similar to the period adaptation (or correction) in previous models (Large and Kolen, 1994; Loehr et al., 2011; Jacoby and Repp, 2012), so that the natural frequency is attracted toward the stimulus frequency. The pulse-like coupling function *f* is used here too so that frequency adaptation is strong when both *θ* and *ϕ* are near zero (Figure 10B).

The second term on the right-hand side of Equation (5) describes a possible mechanism for context-dependent control of tapping frequency during phasing. Here we assume that the tapper tries to maintain a constant rate of relative phase increase, Δ, and also that the tapper can sense the current change rate, $\dot{\psi }=d\psi /dt$. The term applies a positive frequency bias when the current rate is slower than the target rate (i.e., $\dot{\psi }<\Delta$), and the term becomes zero when the current rate matches the target rate. Hence, small Δ would result in gradual phasing with many taps per lap, and large Δ would lead to less gradual phasing with fewer taps per lap. λ is the strength of frequency bias resulting from the intentional control. *g* is a context-dependent gating function that allows different behaviors when beginning and ending phasing by controlling the strength of frequency bias. It is defined as


(7) $$g(\phi ,\theta ,\rho )=\{\begin{array}{ll} 1 & \text{ if }\psi \leq \psi_{\theta }\\ 1-\left|h(\theta ,\rho )\cdot h(-\phi ,\rho )\right| & \text{ if }\psi >\psi_{\theta } \end{array},$$

where *ψ*_*θ*_ is the threshold *ψ* for switching behaviors. When phasing begins, the unwrapped relative phase is near zero and smaller than a threshold, say, *ψ*_*θ*_ = *π*/2. In this case, *g* = 1 so that the second term exerts full frequency bias to facilitate escaping the initial in-phase attractor. Once the relative phase escapes the vicinity of zero (satisfying *ψ*>*ψ*_*θ*_), *g* is made dependent on *ϕ* and *θ* such that *g* gets small (i.e., weak frequency bias) when the tap and the metronome beat are close in time again (i.e., when both *ϕ* and *θ* are near zero, see Figure 10C).^13^ With weak frequency bias, frequency adaptation (the first term in Equation 5) dominates the frequency dynamics, and this allows the oscillator to slow down and end phasing successfully by converging to stable in-phase coordination. Since the gating function is also pulse-like, however, it is possible to overshoot and skip over the in-phase goal if frequency bias is big enough, resulting in unsuccessful phasing.

Note that given the difficulty and self-paced nature of the phasing task, there could be multiple viable strategies for successfully performing phasing. The mechanism of intentional frequency control implemented in Equations (5) and (7) is not proposed as the best or sole mechanism but as one of possible strategies that may be employed by human tappers (see Section 5 General discussion).

#### 4.3.2. Replication of successful, unsuccessful, and incomplete trials

**Figure 11:**
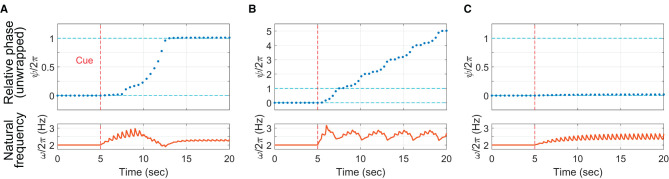
Simulations of the context-dependent model (Equations 4–7): **(A)** successful phasing (Δ = 1 rad/sec), **(B)** unsuccessful phasing (Δ = 3 rad/sec), and **(C)** incomplete phasing (Δ = 0.5 rad/sec). The top plots show the unwrapped relative phase at the time of each “tap” (zero crossing of the wrapped oscillator phase). The bottom plots show the continuous time series of the natural frequency *ω*. Common parameters: *c* = 30, *γ* = 15, λ = 0 (*t* < 5 sec), λ = 3 (*t* ≥ 5 sec), *ψ*_*θ*_ = *π*/2, *ω*_0_ = 4*π* rad/sec (i.e., 2 Hz or 120 BPM), and ρ = 0.7012.

To demonstrate that the context-dependent model can show all three phasing behaviors observed in the human experiment, the model was run for three different values of Δ (the target change rate of relative phase which quantifies the intended gradualness of phasing) while other parameters were fixed. To start each “trial” with in-phase tapping, λ was set to zero for the first 5 s of the simulation and increased to a nonzero constant at *t* = 5 sec (λ = 3 was used for all three simulations). For the intermediate target rate (Δ = 1 rad/sec), the model performed successful phasing by breaking away from the initial in-phase tapping and advancing the relative phase until it reached the in-phase tapping after one phasing lap (Figure 11A). After the cue at 5 s, the natural frequency (the bottom plot) increased gradually (but in a spiky manner due to the pulse coupling) until the relative phase (the top plot) escaped the in-phase attractor (notice the jump around *t* = 8 sec). After that, the natural frequency gradually decreased down to the stimulus frequency (2 Hz) as the relative phase approached and landed on the goal successfully.^14^

For the large target rate (Δ = 3 rad/sec), the model replicated an unsuccessful trial by continuing phasing without stopping after one lap (Figure 11B). The natural frequency decreased when the oscillator was near in-phase with the stimulus (this is when the unwrapped relative phase *ψ* was near an integer multiple of 2*π*), but the frequency bias was still too strong for the frequency adaption to attract the oscillator to in-phase coordination. Finally, with the small target rate (Δ = 0.5 rad/sec), the oscillator failed to escape the attraction of in-phase tapping, replicating an incomplete trial (Figure 11C). The natural frequency increased after phasing started at *t* = 5 sec, but the small Δ did not yield strong frequency bias enough to overcome the frequency adaptation. The simulations show that the strength of frequency bias, controlled by Δ in the model (along with λ), should be just right (not too weak or not too strong) to perform successful phasing. The simulation results for the intermediate and large values of Δ (Figures 11A, B) are consistent with the human experimental finding that phasing was more gradual with more taps per lap in the successful trials than in the unsuccessful trials (Figure 5; see also the next section).

#### 4.3.3. Replication of tempo effect

**Figure 12:**
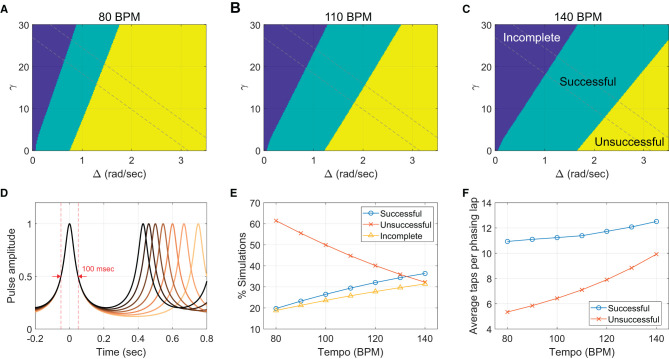
Tempo effect in the context-dependent model. **(A–C)** The classification of model simulations as successful (one phasing lap), unsuccessful (more than one lap), and incomplete (0 lap). For each of seven tempi, 40,401 simulations were run for different combinations of γ and Δ (201 × 201 = 40401). Only the results for the lowest, the middle, and the highest tempi (80, 110, and 140 BPM) are shown. Dashed lines demarcate the anti-diagonal region used for Panels E and F. Parameters: *c* = 30, λ = 0 (before cue), λ = 3 (after cue), *ψ*_*θ*_ = *π*/2, and ρ = 0.7870 (80 BPM), 0.7643 (90 BPM), 0.7425 (100 BPM), 0.7214 (110 BPM), 0.7012 (120 BPM), 0.6817 (130 BPM), and 0.6630 (140 BPM). **(D)** The amplitude of the pulse-like function, |*h*(*θ*, ρ)|, plotted as function of time for different metronome tempi from 80 BPM (the lightest color) to 140 BPM (black). A different ρ was chosen for each tempo (listed above) so that the full width at half max is constant at 100 msec. **(E)** Simulated trial outcomes per metronome condition (only the anti-diagonal area between the dashed lines was counted). **(F)** Average taps per phasing lap in the “successful” and the “unsuccessful” simulations for each tempo.

Next, we tested if the model can replicate the effect of metronome tempo observed in the human experiment. As discussed above (Section 4.1), our hypothesis is that the temporal basin of in-phase attraction (i.e., the temporal range near a metronome beat in which taps are attracted toward the metronome) has a constant width in absolute time regardless of tempo (Repp, 2003, 2004), which results in stronger in-phase attraction at faster tempi. This effect is accounted for in the model by keeping the temporal width of pulse-like function *h* constant across different tempi. This is done by choosing a different ρ for each tempo so that the full width at half max of |*h*| is constant in duration across the tempi (the full width of 100 msec was used in the following simulations; see Figure 12D).^15^

For each metronome tempo used in the human experiment, we ran the context-dependent model for a range of combinations of Δ and γ to sample different model behaviors. γ controls the strength of frequency adaptation and, along with Δ, determines the dynamics of the natural frequency (Equation 5). Previous research on sensorimotor synchronization has shown that while phase correction (phase attraction) is a largely automatic and involuntary process, period correction (frequency adaptation) is in part under voluntary control so that it can be suppressed intentionally (Repp, 2001; Repp and Keller, 2004). Here we assume that untrained participants try different “parameters” for frequency dynamics as they explore and tune their phasing skills. Then, the outcomes of phasing trials during this calibration process could be replicated in simulations with different values of Δ and γ. Here we examine a fixed region of the parameter space (Δ, γ) to obtain the distribution of different phasing outcomes and see if this distribution changes with tempo as found in the human experiment. The coupling strength *c* is fixed in the simulations, assuming phase attraction is not under voluntary control.

Figures 12A–C show the simulation results for the slowest (80 BPM), the middle (110 BPM), and the fastest tempi (140 BPM). For all seven tempi including the three shown in the figure, incomplete phasing was found in the upper left region of the (Δ, γ) space where frequency bias is weak, and frequency adaptation is strong. Unsuccessful phasing was found in the lower right region, with strong frequency bias and weak frequency adaptation. Successful phasing resulted in the region between the incomplete and the unsuccessful regions where frequency bias and frequency adaptation are balanced (see Figure 11 for examples of individual simulations).

An effect of tempo was found in the proportion of different phasing results, which was measured by the sizes of the successful, unsuccessful, and incomplete regions in the (Δ, γ) parameter space (Figures 12A–C). The number of incomplete simulations increased as the tempo increased whereas the number of unsuccessful simulations decreased, both of which correspond to the trends found in the human experiment (Figure 6A). A difference from the human results was also found in that the proportion of successful simulations increased significantly with tempo and occupied more than half of the space at 140 BPM (Figures 12A, C), whereas the human data did not show any clear trend for the successful trials (Figure 6A). The discrepancy may be explained in part by the rectangular region of the (Δ, γ) space chosen for the analysis. Some parts of the region may have not been explored much by the human participants (e.g., where both Δ and γ are very small or very large). A better match with the human data was obtained when only the anti-diagonal region of the parameter space was included in the analysis (demarcated by the dashed lines in Figures 12A–C), which appears to be a more reasonable choice because here the balance between Δ and γ is varied while their sum is maintained comparable. The proportion of successful simulations still increases with tempo (Figure 12E), but all three types have comparable proportions at the fastest tempo as in the human results (compare with Figure 6A). Also, the number of taps per phasing lap in simulations matched the trends in the human data. More taps were made per phasing lap in the successful simulations than in the unsuccessful simulations, and both the numbers increased with tempo (Figure 12F, compare with Figure 6B).

## 5. General discussion

The present study investigated the dynamics of intentional sensorimotor desychronization by analyzing and modeling the experimental data obtained with a novel task of phasing against a metronome. First, the data from an exploratory study (first reported in Hall et al., 2023) were analyzed further to identify the various types of phasing behavior observed in participants who were unfamiliar with the task. Individual trials were categorized as successful, unsuccessful, or incomplete based on the number of phasing laps made in the trial, and the incomplete trials were further categorized into four subtypes. It was found that the number of taps per phasing lap was significantly greater in the successful trials as well as in the participants with higher success rates, suggesting that the gradual increment of relative phase was a key to successful phasing. A strong effect of metronome tempo was found in the proportion of outcome types, with the number of incomplete trials increasing with the increase of tempo while the number of unsuccessful trials decreased with tempo increase. It was also found that phasing was more gradual at fast tempi, with more taps per phasing lap. A dynamical systems interpretation of the results was given, which characterizes phasing performance as an intentional goal-directed action under the constraints of intrinsic attractor dynamics (i.e., the presence of in-phase attractor). Based on this view, a minimal dynamical model was presented which captures the interaction of intrinsic constraints and intentional control in a periodically forced oscillator with context-dependent frequency dynamics. It was shown that the single model can produce all three types of phasing behavior as well as replicate the tempo effect observed in the human experiment, supporting the dynamical systems explanation of phasing performance.

The present study demonstrated that the musical technique of phasing offers a unique setup for investigating human rhythmic behavior. Phasing involves desynchronization from a sensory stimulus. Thus, the processes underlying phasing performance cannot be described as error correction, the mechanism commonly attributed to sensorimotor synchronization (Mates, 1994; Jacoby and Repp, 2012). Also, intentional desynchronization in phasing is different from the experimental tasks that require participants to intentionally ignore distractor tones (Repp, 2003, 2004) or to suppress period correction after a tempo change (Repp and Keller, 2004). Phasing requires a controlled manipulation of the relative phase between the motor output (taps) and the sensory stimulus (metronome beats). Therefore, participants cannot perform successful phasing if they intend to ignore or suppress the stimulus entirely. Gradual desynchronization during phasing is also distinct from the intentional switching of coordination modes studied in the coordination dynamics literature which requires an instant change of behavioral patterns (Scholz and Kelso, 1990; Serrien and Swinnen, 1999).

The present study showed that it is plausible to describe the dynamics of phasing performance in terms of the interaction between the intrinsic stability of in-phase synchronization and the task-specific intention of desynchronization. Dynamical systems theory provides the right language to describe such cooperation and competition among multiple underlying constraints as well as the pattern of behaviors emerging lawfully from such interactions (Beek et al., 2000; Temprado and Salesse, 2004). In the proposed model of phasing performance (Equations 4–5), the intrinsic dynamics (the stability of in-phase coordination) and the intentional control (context-dependent frequency bias) are expressed as distinct terms in the differential equations, and their collective dynamics determine the behavior of the model. The dynamical systems approach presented in this study is not limited to music performance but could be applied to intentional desynchronization in other areas of human coordination such as sports, conversation, and crowd behavior (Passos et al., 2016; Paxton and Dale, 2017; Warren et al., 2023).

The context-dependent phasing model presented here is a continuous-time model described by two differential equations (Equations 4–5). The present continuous-time model can be compared to previous discrete-time models of rhythmic entrainment described by the difference equations for phase attraction and period adaptation (Large and Kolen, 1994; Large and Jones, 1999). More recently, the frequency adaptation in continuous-time oscillators was analyzed (Righetti et al., 2006) and studied as a model of the perception of musical rhythms (Lambert et al., 2016). The present model can be considered equivalent to these models (in qualitative dynamics) if the frequency equation included only the frequency adaptation term. Thus, the construction of the present model can be understood as a new term for intentional frequency control added to the general intrinsic dynamics underlying the synchronization and frequency adaptation to an external rhythm. Such synergistic combination of intrinsic and task-specific dynamics is also found in other models of rhythmic coordination. For example, the process of learning a new specified phase of bimanual coordination (other than in-phase and antiphase, e.g., 90°) can be modeled by introducing additional terms that stabilize the new phase to a model describing the intrinsic dynamics of bimanual coordination, such as the Haken–Kelso–Bunz model (Haken et al., 1985) for which in-phase and antiphase modes are stable (Schöner and Kelso, 1988b; Schöner et al., 1992).

In this paper, a continuous-time model was used to describe the dynamics of phasing performance, but the same qualitative dynamics should be achievable with a discrete-time model because most interactions in the model (e.g., coupling and frequency adaptation) are temporally confined. To capture the discrete nature of the stimulus (metronome beats) and the motor output (taps), a new analytic pulse-like function *h* was introduced which varies the width with the parameter ρ (see Appendix). This allowed replicating the tempo effect by keeping the pulse width constant in absolute time. It was also shown that the coupling function *f*, which is a product of two *h* functions (Equation 6), can vary the form continuously between the sine coupling (used in standard continuous-time phase models) and the impulse coupling (equivalent to the coupling in discrete-time models). Hence, the analytic form of the present model can be used to understand the relation and transition between continuous-time and discrete-time models, which is left for future studies.

The context-dependent frequency control in the present model is a simple mechanism that is in no sense intended as the best or only strategy for phasing performance. It was assumed that the current change rate of relative phase is available to the tapper and that the tapper tries to maintain a constant rate of change. With this mechanism, the model can produce all three main types of phasing behavior, but not all subtypes of the incomplete phasing. The present model cannot produce the halfway subtype because it does not have an attractor at the antiphase relation (*ψ* = ±*π*). As in the Haken–Kelso–Bunz model (Haken et al., 1985), the antiphase attractor can be introduced by adding higher-order coupling terms to the model. With the antiphase attractor, the model would also show the slowdown of phasing near the antiphase relation which was observed in some trials in the human experiment (see the plateau of relative phase near *ψ*/(2*π*) = 0.5 in Figure 4A). These modeling possibilities, however, are beyond the scope of the present paper and will be studied elsewhere. The backward subtype of incomplete trials, in which the relative phase decreased over time, suggests that the participants were sometimes confused about the direction of phasing. This behavior is not possible in the present model because it assumes that the tapper knows the current change rate of relative phase. To replicate the backward behavior, this assumption must be relaxed by making the information available on the current state less accurate or partial (e.g., with stochastic noise). Such possibilities could be explored in future research to study the initial stages of learning phasing performance as a new skill.

Here we focused our discussion on in-phase and antiphase coordination, but it is known that humans can produce rhythms at other phases as well. Both musicians and nonmusicians were shown to be capable of producing rhythms made of two unequal intervals, with produced rhythms attracted to a ratio near 1:2 or 120° phase (Repp et al., 2011, 2012). Also, it was shown that bimanual coordination in 90° can be learned by training with pacing signals (Zanone and Kelso, 1992). These coordination phases may be related to different ratios of multifrequency coordination, for example, 120° coordination related to 1:3 frequency ratio (triple subdivision), and 90° coordination to 1:4 frequency ratio (quadruple subdivision; Dotov and Trainor, 2021). As dynamical systems analysis indicates (deGuzman and Kelso, 1991; Haken et al., 1996; Kim and Large, 2019), and both perception and production studies showed (Razdan and Patel, 2016; Mathias et al., 2020), these coordination phases are less stable than in-phase and antiphase relations, which may be related to the simple frequency ratios of 1:1 and 1:2 respectively. Due to low dynamic stability (especially in the presence of noise inherent to motor movements), the attraction toward phases other than in-phase and antiphase may not be clearly observed during phasing performance, where intentional control competes with stronger attractors. One way to study the attraction at other phases is to measure the stability of sensorimotor coordination at a set of fixed phases in separate trials (Yamanishi et al., 1980; Tuller and Kelso, 1989; Dotov and Trainor, 2021). Our ongoing study involving finger tapping to a metronome at a specified phase suggested the presence of weaker attractors near ±120° as well as stronger attractors at in-phase (0°) and antiphase (180°) relations.

The present study adds to the growing body of research that employs dynamical systems theory to study music performance (Demos et al., 2014; Schiavio et al., 2022; Tichko et al., 2022). Early oscillator models focused on the perception and production of musical rhythms in individuals (Large and Kolen, 1994; Large and Palmer, 2002; Loehr et al., 2011). More recently, dynamical systems analysis and modeling has extended to the interpersonal coordination in musical dyads and ensembles (Demos et al., 2019; Heggli et al., 2019; Roman et al., 2019; Bégel et al., 2022; Dotov et al., 2022). The present study, motivated by a case study of phasing between two expert musicians (Hartenberger, 2016; Schutz, 2019), aimed to uncover the underlying dynamics of phasing performance by studying non-expert participants with a simpler task of phasing against a metronome. Both successful and failed attempts at controlled phasing informed the identification of underlying dynamics and the construction of a minimal dynamical model. In contrast, the first model developed for the expert phasing data was not able to miss the target (see text footnote 2), although this can also happen to professional musicians (Hartenberger, 2016). A next step in this research would be to study interpersonal phasing systematically in controlled experiments, with both expert musicians and non-experts. Future studies could explore the manipulation of sensory feedback and informational coupling (Rosso et al., 2021, 2022) and the use of a virtual agent (governed by a dynamical system) as a more controlled phasing partner (Kelso et al., 2009; Van Kerrebroeck et al., 2021). The findings of the present study will guide the design and analysis of future experiments and modeling works.

The dynamical systems account presented in this paper provides an intuitive explanation of what happens in phasing performance. We described the dynamics of phasing performance in terms of the cooperative and competitive interactions of different underlying forces (intrinsic dynamics and intentional control). Musicians and music scholars have long described the experience of music listening and performance in terms of dynamic qualities and forces (Zuckerkandl, 1956; Larson, 2012). For example, when we listen to a tonal melody, we feel the attraction of the leading tone toward the tonic (Lerdahl, 1996). Russell Hartenberger, one of the expert percussionists in the case study, described in detail the forces of attraction and resistance he experienced in phasing performance (Hartenberger, 2016). The dynamical systems account suggests that these subjective and bodily experiences are not merely metaphors but actual forces involved in music making which can be quantitatively measured and mathematically formalized. At the same time, dynamical systems theory provides conceptual tools that can help musicians to understand and describe their subjective experience (Schiavio et al., 2022). An intuitive understanding of performance dynamics may provide musicians with new insights that can enrich their musical experience and help with performance and practice strategies.

## Data availability statement

Publicly available datasets were analyzed in this study. This data can be found here: https://osf.io/4uhtb/.

## Ethics statement

The studies involving humans were approved by the UConn-Storrs Institutional Review Board (IRB). The studies were conducted in accordance with the local legislation and institutional requirements. The participants provided their written informed consent to participate in this study.

## Author contributions

JK conceived and designed the study, analyzed the data, created the models, and wrote the manuscript.

## Figures and Tables

**Figure A1:**
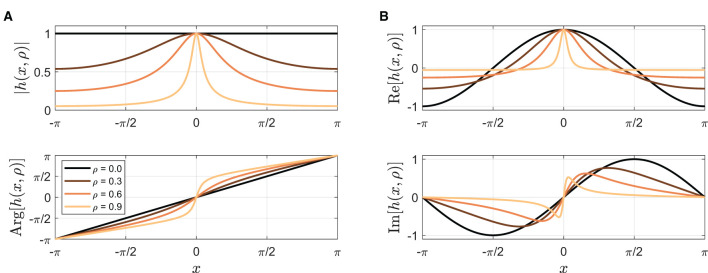
**(A)** The amplitude and phase, and **(B)** the real and imaginary parts of the pulse function *h*(*x*, ρ) for ρ= 0, 0.3, 0.6, and 0.9.

## Footnotes

^1^Kim, J. C. (manuscript in preparation). Push and pull: The dynamics of phasing performance in Steve Reich's music.

^2^The dynamical systems analysis and modeling of the Drumming data was first reported in Kim (2019).

^3^The direction of phasing (getting ahead of the metronome, instead of getting behind) follows the original phasing process in Reich's *Drumming* in which Drummer 2 places each note increasingly ahead of Drummer 1 who tries to hold a constant tempo. Phasing in the opposite direction (i.e., by falling behind a metronome) may prove more difficult because it would be harder to break away from in-phase synchronization when the metronome precedes the tap. This would make an interesting follow-up study.

^4^Note that relative phase defined this way has an opposite sign from asynchrony commonly used in the sensorimotor synchronization literature. Relative phase, favored in dynamical systems research, is based on system states, while asynchrony is based on event times. When the tap is ahead of the metronome, the tapper's relative phase to the metronome is positive because the tapper's state (phase) is more advanced than the metronome's state at a given moment in time. The asynchrony is negative in this situation because the tap time is earlier than the metronome tick time.

^5^This was done by subtracting the minimum initial relative phase from the maximum unwrapped relative phase reached in the trial, dividing the result by 2*π*, and then applying the floor function.

^6^The classification of successful, unsuccessful, and incomplete trials was initially reported in the original paper (Hall et al., 2023). All other results described here are new.

^7^The incomplete trials are not included in this analysis because taps per lap could not be computed when no complete lap was made.

^8^Intrinsic stability means that a pattern is stable and occurs spontaneously in the absence of specific task requirements (Schöner and Kelso, 1988a). Task-specific intention interacts with the intrinsic dynamics of the system and alters the attractor layout, inducing behavioral change.

^9^“Phase” refers to the angular component of oscillation, not to be confused with “phasing” which refers to the act of varying the phase relation between patterns.

^10^The wrapped phase is obtained by mapping the original unwrapped phase onto the range from −*π* to *π* by adding or subtracting a multiple of 2*π*.

^11^When Δ*ω* = *c*, the *d*ψ**/*dt* curve touches the *ψ* axis at *π*/2 or 90° (imagine a case between Figures 9B, C). In this case, there is a half-stable fixed point at *ψ* = *π*/2: it is stable when approaching from below (lower than *π*/2) but unstable from above (higher than *π*/2). Thus, when the relative phase is perturbed upward towards *π* (e.g., by stochastic noise), it would keep increasing until it stops at the half-stable point, completing only one phasing lap. However, phasing in this system ends at *ψ* = *π*/2, not at *ψ* = 0 (in-phase) as required in the experiment. Thus, this system is implausible as a model of phasing performance.

^12^Figure 10B is the two-dimensional vector field for *ψ* when *ω* = *ω*_0_, which can be compared to the one-dimensional vector field in Figure 9A.

^13^Both the coupling function *f* and the gating function *g* are based on the same complex pulse function *h*(*θ*, ρ)·*h*(−*ϕ*, ρ), but the former uses the imaginary part while the latter uses the absolute value. See Figure A1 in Appendix.

^14^Notice that after the model ended phasing successfully at around *t* = 13 sec, the natural frequency increased back by a small amount (Figure 11A). This is because the pulse-like gating function *g* does not suppress frequency bias completely even when the oscillator is stably entrained in phase with the stimulus: *g* is zero only at the time of taps (phase zero-crossing) and nonzero at other times (see Figure 10C, along the diagonal). In this simulation, frequency bias is suppressed enough so that frequency adaptation can keep the oscillator in phase with the stimulus. To model the switch of intention after reaching the goal, we could introduce another mechanism that, for example, eliminates frequency bias after the relative phase stops advancing at the intended goal. To keep the model minimal, however, we did not explore such additional features here.

^15^For a fixed ρ, the temporal width of *h* is proportional to the metronome period (i.e., it is wider at slow tempi) because *h* is a function of phase, not of time (see Appendix). When the pulse width is fixed in measures of *time*, it is wider at faster tempi in measures of *phase*.

## Appendix

The pulse-like function *h* is a 2*π*-periodic complex analytic function of phase *x* defined as

(8)
$$\begin{array}{l} \begin{array}{c} h\left(x,\rho \right)=\frac{\left(1-\rho \right)e^{\text{i}x}}{1-\rho e^{\text{i}x}}\\ =\left(1-\rho \right)\left(e^{\text{i}x}+\rho e^{2\text{i}x}+\rho^{2}e^{3\text{i}x}+\cdots \right), \end{array} \end{array}$$

where 0 ≤ ρ < 1 is a parameter controlling the width of the pulse (Figure A1), and i is the imaginary unit. It is an infinite series containing a complex sinusoid *e*^i*x*^ and its harmonics. The normalization factor (1−ρ) is multiplied so that the maximum amplitude |*h*(0, ρ)| is constant regardless of ρ. The *n*-th harmonic has a coefficient proportional to ρ^*n*−1^, so the strength of high-order harmonics depends on ρ. On one extreme, *h* becomes a sinusoid when ρ = 0 (only the fundamental survives). On the other end, *h* approaches the Kronecker delta function (an impulse function) when ρ approaches 1. Between the two extremes, the shape of *h* changes continuously between a perfect sinusoid and a discrete impulse, depending on ρ (Figure A1).

As mentioned in Section 4.3, the pulse coupling *f* in Equation (4) is a general coupling function that includes the sine coupling in Equation (2) as a special case. Since *h*(*x*, 0) = *e*^i*x*^,

(9)
$$\begin{array}{l} \begin{array}{c} f\left(\phi ,\theta ,0\right)=\text{Im}\left[h\left(\theta ,0\right)\cdot h\left(-\phi ,0\right)\right]\\ =\text{Im}\left[e^{\text{i}\left(\theta -\phi \right)}\right]\\ =\sin\left(\theta -\phi \right). \end{array} \end{array}$$

This shows that Equation (4) becomes Equation (2) when ρ = 0.
